# Toll-Like Receptors are critical in controlling colonic inflammation and cancer

**Published:** 2011-01-10

**Authors:** Hui Xiao, Weiguo Yin, Mohammed A Khan, Muhammet F Gulen, Bruce A Vallance, Xiaoxia Li

**Affiliations:** 1 Unit of Immune Signaling and Regulation, Institut Pasteur of Shanghai, Shanghai 200025, PR China; 2 Department of Immunology, Cleveland Clinic Foundation, Cleveland, OH 44195, USA; 3 Division of Gastroenterology, University of British Columbia and BC Children’s Hospital, Vancouver, BC V6T 1Z4, Canada

## 

Despite the presence of large number and diverse populations of commensal microbes, gut mucosa has evolved to maintain “microbial-tolerance”, which is critically regulated by well-controlled Toll-like receptor (TLR) signaling. Deregulated TLR signaling has been linked to the pathogenesis of inflammatory bowel disease and colon cancer; however, the underlying mechanisms need to be further defined. In this study, we uncovered that lack of SIGIRR, a negative regulator for TLR and IL-1R signaling, led to increased genetic instability and LOH of *Apc*, resulting in spontaneous colonic polyposis in *Apc^min/+^/Sigirr^-/-^* mice. Importantly, elevated colonic tumorigenesis in *Apc^min/+^/Sigirr^-/-^* mice is dependent on the presence of commensal microbes in gut, implicating a critical role for TLR signaling in tumorigenesis. Furthermore, we demonstrated that SIGIRR-modulated TLR-mediated tumor initiation is mainly through the activation of the Akt-mTOR axis, which promotes cell cycle progression through its impact on posttranscriptional control of the key cell cycle regulators (Cyclins, c-Myc and cdk2). Moreover, abrogation of mTOR pathway by rapamycin prevented microadenoma and polyps formation in *Apc^min/+^/Sigirr^-/-^* mice, providing new insights into treating human cancers. In addition, augmented production of proinflammatory cytokines, such as IL-6 and IL-23, further promoted tumor growth in *Apc^min/+^/Sigirr^-/-^* mice. Epithelium specific re-expression of SIGIRR in *Apc^min/+^/Sigirr^-/-^* mice ameliorated intestinal tumorigenesis. In summary, this study indicates that SIGIRR is a critical tumor suppressor that controls tumorigenesis by inhibiting TLR-induced mTOR and NFkB pathways in colonic epithelium.

